# Self-reported health as a predictor of mortality: A cohort study of its relation to other health measurements and observation time

**DOI:** 10.1038/s41598-020-61603-0

**Published:** 2020-03-17

**Authors:** Geir Lorem, Sarah Cook, David A. Leon, Nina Emaus, Henrik Schirmer

**Affiliations:** 1 UiT The Arctic University of Norway, Tromsø, Norway; 2 London School of Hygiene & Tropical Medicine, London, United Kingdom; 3 University of Oslo, Oslo, Norway; 4 Akershus University Hospital, Nordbyhagen, Norway

**Keywords:** Diseases, Epidemiology, Risk factors

## Abstract

Self-reported health (SRH) is widely used as an epidemiological instrument given the changes in public health since its introduction in the 1980s. We examined the association between SRH and mortality and how this is affected by time and health measurements in a prospective cohort study using repeated measurements and physical examinations of 11652 men and 12684 women in Tromsø, Norway. We used Cox proportional hazard regression to estimate hazard ratios (HRs) of death for SRH, controlling for pathology, biometrics, smoking, sex and age. SRH predicted mortality independently of other, more objective health measures. Higher SRH was strongly associated with lower mortality risk. Poor SRH had HR 2.51 (CI: 2.19, 2.88). SRH is affected by disease, mental health and other risk factors, but these factors had little impact on HRs (Poor SRH: HR 1.99; CI: 1.72, 2.31). SRH predicted mortality, but with a time-dependent effect. Time strongly affected the hazard ratio for mortality, especially after ten-year follow-up (Poor SRH HR 3.63 at 0–5 years decreased to HR 1.58 at 15–21 years). SRH has both methodological and clinical value. It should not be uncritically utilised as a replacement instrument when measures of physical illness and other objective health measures are lacking.

## Introduction

The connection between self-reported health (SRH) and mortality was described quite early in the history of SRH. The predictive validity of SRH has repeatedly been confirmed and even found to be a stronger predictor of all-cause mortality than instruments designed specifically for this purpose^1^. SRH has also been shown to be stable across cultures, communities and different age groups^2–9^. SRH is today frequently used as the only measure of health when more extensive measurements of health are lacking.

SRH is related to disease burden as well as to mental health and social context. SRH is known to be an inclusive instrument that seems to go beyond a model of health as an absence of disease. SRH reflects coping resources and influences health-related behaviour that affects outcome^10–17^. It is therefore plausible that SRH predicts mortality because subjects merely report their clinical and subclinical conditions^18,19^. SRH is also a dynamic instrument likely to change during a life span, but we find no studies that examine the effect of observation time on its ability to predict mortality.

In this paper, we ask whether SRH still represents a valid instrument of epidemiological research. Studies of SRH and mortality often lack comprehensive health measures, are based on limited samples (e.g. patient populations) or have limited follow-up time. The Tromsø study (TS) included measures of SRH in 1986. We are thus able to track how subjects in the same population rated their health on three different occasions over 25 years as they aged and the public health context changed around them. We aim to determine the strength of association between different levels of SRH and mortality risk, how this is affected by other measurements of health, and whether these associations remain stable over time.

## Results

### Changes in characteristics from 1994 to 2007

Table 1 shows the characteristics of the cohort during the three surveys. We see that more subjects lived with a higher number of comorbid diseases as they grew older. The mean age increased from 47.8 in 1994 to 62.3 in 2001, falling slightly to 61.2 in 2007. The comorbid burden has been measured using the Health Impact Index (HII), and Table 1 shows how age- and sex-standardised mean HII increased from 1.2 in 1994 to 1.6 in 2007. We see a corresponding decline in SRH over the same period. In 1994 70.4% reported SRH as good or very good, but the figure declined to 61.1% in 2001 and 63.9% in 2007. The percentage of participants who rated their health as very good fluctuated; here, the figures were 14.7% in 1994, 7.9% in 2001 and 13.4% in 2007. Those with self-reported poor health remained stable at 2.3% in both 1994 and 2001, but increased to 5.5% in 2007. Cholesterol and blood pressure increased during the follow-up period but, as shown in the age-adjusted estimates, there was a relatively small but statistically significant decline over the period. There was an increase in obese subjects (BMI ≥ 30 kg/m^2^) from 11.2% in 1994 to 19.6% in 2007, while the number of daily smokers decreased from 35.7% to 20.1% in the same period. We found that all trends were significant (p < 0.0001). All pairwise comparisons were significant, and the effect sizes were moderate.

**Table 1:** Characteristics of the cohort in the Tromsø 4–6 surveys with age- and sex-standarised estimates.

	1994			2001			2007		
	Count	Freq%	Adjusted%	Count	Freq%	Adjusted%	Count	Freq%	Adjusted%
**Self-reported health**									
Poor	561	2.3%		157	2.3%		509	5.5%	
Not so good	6631	27.2%		2445	36.5%		2819	30.6%	
Good	13567	55.7%		3563	53.2%		4665	50.6%	
Very good	3577	14.7%		530	7.9%		1234	13.4%	
**Age and sex**									
Age (Mean/SD)	47.8	(14.7)		62.6	(11.4)		61.2	(11.1)	
Women	12684	52.1%		3858	56.6%		4967	53.3%	
Men	11652	47.9%		2958	43.4%		4349	46.7%	
	**Mean**	**SD**	**Adjusted**	**Mean**	**SD**	**Adjusted**	**Mean**	**SD**	**Adjusted**
**Disease and risk factors**									
Comorbid disease (Mean/SD)	1.0	(1.7)	1.2	1.8	(2.2)	1.4	1.8	(2.2)	1.6
Hypertension (Mean/SD)									
Systolic blood pressure (mmHg)	135.2	(20.6)	139.4	140.8	(21.9)	135.1	138.9	(23.4)	136.0
Diastolic blood pressure (mmHg)	78.5	(12.4)	80.4	80.9	(12.3)	79.7	78.3	(10.7)	*77.9*
Hyperlipidia (Mean/SD)									
Total cholesterol (mmol/l)	6.1	(1.3)	6.3	6.3	(1.2)	6.1	5.7	(1.1)	5.6
High density lipoprotein cholesterol (mmol/l)	1.5	(0.4)	1.5	1.5	(0.4)	1.4	1.5	(0.4)	1.5
	**Count**	**Freq%**	**Adjusted%**	**Count**	**Freq%**	**Adjusted%**	**Count**	**Freq%**	**Adjusted%**
Mental health symptoms (HSCL-10									
No symptoms	2046	8.4%	8.1%	2243	38.0%	38.9%	2945	33.6%	33.6%
Some symptoms	15630	64.2%	64.0%	2309	39.1%	38.1%	3426	39.1%	38.1%
Sub-threshold	4918	20.2%	20.5%	984	16.6%	17.0%	1703	19.4%	20.4%
Significant symptoms	1742	7.2%	7.4%	374	6.3%	6.0%	688	7.9%	7.9%
Body Mass Index									
<18.5 Kg/m^2^	307	1.3%	1.3%	71	1.0%	0.6%	64	0.7%	0.6%
18.5–25 kg/m^2^	12336	50.7%	49.3%	2405	35.5%	39.6%	3145	33.8%	36.0%
25–30 kg/m^2^	9082	37.3%	38.1%	2991	44.1%	44.2%	4191	45.1%	43.7%
>30 kg/m^2^	2611	10.7%	11.2%	1312	19.4%	15.6%	1902	20.4%	19.6%
Smoking status									
Current smoker	8844	36.4%	35.7%	1863	27.5%	32.4%	1739	19.0%	20.1%
Previous smoker				2594	38.3%	32.4%	4112	44.9%	40.9%
Never smoked	15481	63.6%	64.3%	2317	34.2%	35.3%	3309	36.1%	38.9%

^a^The cross-tabulations between time and self-reported health (Pearson’s chi2(6) = 612.9177 P < 0.001), Sex (Pearson’s chi2(2) = 43.0865 P < 0.001) and Smoking (Pearson’s chi2(4) = 1.2e + 04 P < 0.001) show that the differences are statistically significant at an alpha level of 5%.

^b^The variances between the different surveys were checked with ANOVA for age. F(2) = 5329.73, p < 0.001; Comorbid disease: F(2) = 829.79, p < 0.001; Sys. BP: F(2) = 229.84, p < 0.001; Dias. BP: F(2) = 120.50, p < 0.001; Total cholesterol: F(2) = 504.50, p < 0.001; HDL: F(2) = 38.99, p < 0.001; BMI: F(2) = 788.27, p < 0.001.

Table 2 shows the observed and age-adjusted values versus SRH at baseline. The analytical goal was to show how age, comorbidity and underlying risk factors were associated with SRH. There was a significant positive association between SRH and beneficial risk factors. All surveys showed a similar trend, but the table only displays the baseline estimates. SRH levels declined with increasing age. SRH was strongly associated with both mental health symptoms and specific medical conditions (HII). Age-adjusted SRH levels were consistently associated with beneficial levels of BP, cholesterol and BMI. There was a large and independent effect size for age, comorbid disease and mental health symptoms and a moderate to small effect size for the other risk factors. Despite being a subjective and immediate assessment of overall health, SRH was associated with other, more objective health measures.

**Table 2:** Observed values versus self-rated health at baseline.

	Age		Comorbid disease (HII)				Systolic blood pressure/mmHg				Diastolic blood pressure/mmHg				Total cholesterol/mmol/l			
	Mean	SE	Crude mean	SE	Adjusted mean	SE	Crude mean	SE	Adjusted mean	SE	Crude mean	SE	Adjusted mean	SE	Crude mean	SE	Adjusted mean	SE
**Self-reported health**																		
*Poor*	59.59	0.66	2.85	0.13	1.91	0.10	141.55	1.03	133.79	0.85	80.75	0.57	78.30	0.61	6.40	0.06	6.11	0.05
*Not so good*	54.97	0.18	1.76	0.03	1.41	0.02	140.41	0.29	134.73	0.22	81.22	0.16	78.75	0.15	6.44	0.02	6.17	0.02
*Good*	45.89	0.12	0.70	0.01	0.74	0.01	134.11	0.17	135.69	0.15	78.20	0.10	78.79	0.10	6.02	0.01	6.09	0.01
*Very Good*	39.71	0.19	0.37	0.01	0.42	0.02	128.48	0.26	133.46	0.40	74.41	0.18	77.16	0.25	5.65	0.02	5.98	0.03
**P-value**	<0.001		<0.0001		<0.0001		<0.0001		<0.0001		<0.0001		<0.0001		<0.0001		<0.001	
			**HDL/mmol/l**				**Mental health**				**BMI/kg/m**^**2**^				**Daily smokers**			
			**Crude mean**	**SE**	**Adjusted mean**	**SE**	**Crude mean**	**SE**	**Adjusted mean**	**SE**	**Crude mean**	**SE**	**Adjusted mean**	**SE**	**Smokers**	**Non-smokers**		
**Self-reported health**																		
*Poor*			1.49	0.02	1.48	0.02	2.06	0.03	2.09	0.04	25.79	0.22	25.38	0.28	41.9%	58.1%		
*Not so good*			1.50	0.01	1.48	0.01	1.70	0.01	1.74	0.01	25.91	0.05	25.57	0.06	39.6%	60.4%		
*Good*			1.50	0.00	1.51	0.00	1.47	0.00	1.46	0.00	25.14	0.03	25.20	0.03	36.9%	63.1%		
*Very Good*			1.53	0.01	1.55	0.01	1.30	0.00	1.29	0.01	24.21	0.05	24.66	0.07	27.3%	72.7%		
**P-value**			<0.0001		<0.0001		<0.0001		<0.0001		<0.0001		<0.0001		<0.0001			

ANOVA confirms that there is a significant (p < 0.0001) effect of SRH for all risk variables. Test results were for age F(3) = 1259.02, p < 0.0001, ω = 0.13; Comorbid disease F(3) = 1102.58, p < 0.0001, ω = 0.12; Total cholesterol F(3) = 549.2, p < 0.0001, ω = 0.04; HDL F(3) = 549.2, p < 0.0001, ω = 0.04; Systolic blood pressure F(3) = 310.7, p < 0.0001, ω = 0.04; Diastolic blood pressure F(3) = 253.3, p < 0.0001, ω = 0.03.

### Mortality hazard ratios in relation to self-reported health

**Figure 1:**
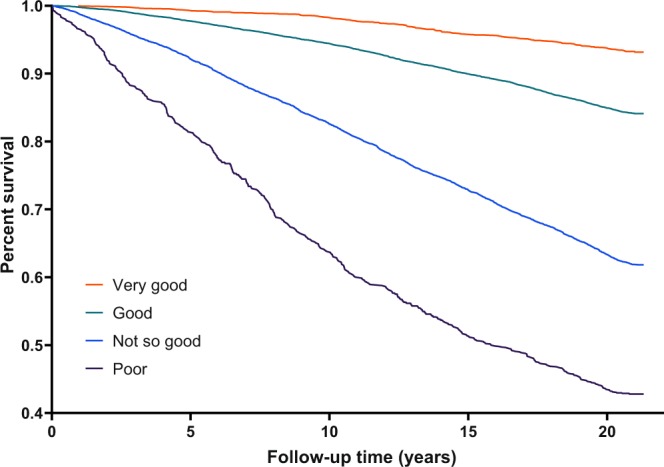
Kaplan-Meier survival estimates for different levels of self-reported health. The survival curve grouped by self-reported health categories in Tromsø 4 (1994).

A total of 7662 of 24336 subjects died between study entry in 1994 and the end of the study. The average follow-up time was 21.1 years. Among 1126 subjects with poor SRH, 537 died, providing an incidence rate of 0.043 per person-year. In contrast, of 4433 subjects with very good SRH, 346 died. The incidence rate for those with very good SRH (0.0043 per person-year) is therefore one-tenth of those with poor SRH. Figure 1 shows how the Kaplan-Meier survival estimates spread out over the levels of SRH.

A Cox proportional hazard model was used to determine the hazard ratios associated with lower levels of SRH. Table 3 shows the distribution and adjusted hazard ratios using baseline health metric scores and updated repeated scores as time-dependent covariates. Very good was the reference category since we expected it to be associated with the highest level of resilience towards mortality. The univariate estimates control for cohort and sex, while the adjusted ones are fully fitted for all variables. We see that higher levels of SRH were strongly associated with lower mortality risk. The univariate model indicates that the hazard ratio decreased in approximately equal increments from very good to poor. Poor SRH had a hazard ratio for all-cause mortality of HR 2.51 (CI: 2.19, 2.88), while not so good SRH had HR 1.94 (CI: 1.73, 2.17) as compared to very good SRH in the fully fitted model. Good SRH was associated with a 36% increased risk (HR 1.36 CI: 1.22, 1.52). This implies that the risk of all-cause mortality associated with reporting the lowest level of SRH is approximately two and a half times as high as the reference category.

**Table 3:** Distribution and adjusted hazard ratios for self-reported health, comorbid disease, mental health, and biometric scores using baseline health metric scores and updated repeated scores as time-dependent covariates.

	No. of observations	Person-time (years)	Died	Rate	Hazard ratio (univariate)	LLCI	ULCI	Hazard ratio (adjusted)	LLCI	ULCI
**Self-Reported Health**										
Poor	1126	12349	537	0.043	2.51	2.19	2.88	1.99	1.72	2.31
Not so good	8918	130490	3620	0.028	1.94	1.73	2.17	1.69	1.51	1.90
Good	15694	290892	3159	0.011	1.36	1.22	1.52	1.28	1.15	1.43
Very good (Baseline)	4433	80484	346	0.0043	1.00			1.00		
**Age and sex**										
Cohort (Year born)	24336	514214	7662	0.015	0.89	0.89	0.90	0.90	0.89	0.90
Women (Baseline)	12684	271367	3744	0.014	1.00			1.00		
Men	11652	242847	3918	0.016	1.54	1.47	1.61	1.69	1.61	1.77
**Disease and risk factors**										
Comorbid disease	24336	514214	7662	0.015	1.05	1.04	1.06	1.03	1.02	1.04
Hypertension										
*Normal blood pressure (baseline)*	6895	128111	711	0.006	1.00			1.00		
*Sub-threshold*	11982	208321	1776	0.009	0.91	0.84	1.00	0.94	0.86	1.03
*Hypertension*	11046	177783	5175	0.029	0.98	0.91	1.07	1.08	0.99	1.18
Hyperlipidaemia										
*Ideal values (Baseline)*	5096	95028	627	0.007	1.00			1.00		
*Sub-threshold*	19315	365522	5427	0.015	0.94	0.86	1.02	0.96	0.88	1.04
*Hyperlipidaemia*	3938	53664	1608	0.030	1.05	0.95	1.15	1.04	0.95	1.15
Mental health group										
*No symptoms (Baseline)*	5807	82096	1115	0.014	1.00			1.00		
*Some symptoms*	17075	292491	4182	0.014	1.27	1.19	1.36	1.14	1.07	1.22
*Sub-threshold*	6598	102174	1647	0.016	1.40	1.30	1.51	1.12	1.03	1.21
*Significant symptoms*	2460	37453	718	0.019	1.62	1.47	1.78	1.13	1.02	1.25
BMI group										
<*18.5 kg/m*^*2*^	377	5575	172	0.031	1.94	1.66	2.26	1.59	1.36	1.86
*18.5–25 kg/m*^*2*^ *(baseline)*	12932	247198	3012	0.012	1.00			1.00		
*25–30 kg/m*^*2*^	11365	196558	3128	0.016	0.88	0.84	0.92	0.91	0.87	0.96
>*30 kg/m*^*2*^	3963	64883	1350	0.021	0.98	0.92	1.05	0.98	0.92	1.05
Smoking status										
*Current smoker (baseline)*	9132	171827	2520	0.015	1.00			1.00		
*Previous*	5190	63165	1188	0.019	0.48	0.44	0.51	0.47	0.44	0.51
*Never smoked*	15540	279222	3954	0.014	0.59	0.56	0.62	0.62	0.59	0.65

HR = Hazard ratio, LLCI = Lower level 95% confidence interval, ULC I = Upper level 95% confidence interval, Number of participants = 24336, deaths = 7662, time at risk = 514214.4 person years, LR chi2(16) = 14125.79, p < 0.0001. Note: Variables in time-varying covariates equation interacted with time. Univariate model is sex- and age-adjusted.

We controlled for all the adjusted hazard ratios. We see that the adjusted HRs for the SRH categories are slightly lower, but the univariate HRs concur with the univariate model. Comorbid disease, hypertension, hyperlipidaemia, body mass, and smoking are all associated with a significant hazard ratio, but the relationship between all-cause death and hypertension and hyperlipidaemia is not significant in the fully fitted model. Despite the association (Table 2) between SRH and more objective health measures, we still regard the attenuation as very small when we control for these factors.

### Time affected the hazard ratio for mortality

**Figure 2:**
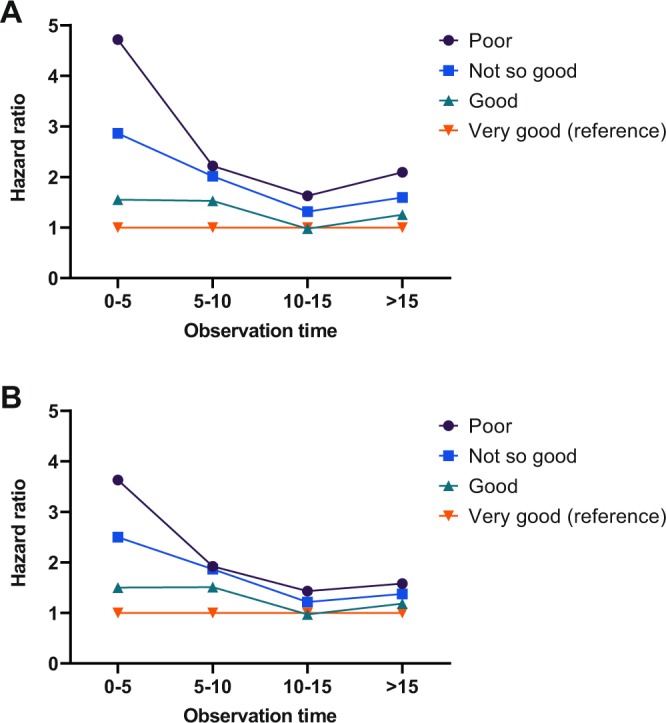
Development of hazard ratio over time. (**A**) All hazard ratios are controlled for age and sex. (**B**) Adjusted mortality risk also controls for pathology and other risk variables.

SRH is a subjective assessment of current health status. This implies that the evaluation is likely to change with age, onset of illness, or when other health-related factors change. Table 4 and Fig. 2 show SRH for the Tromsø 4 cohort without updated values at five-year intervals. The aim of the analysis was to examine how the HRs changed during the time since SRH was rated in 1994. We see how the HRs decreased in the same cohort when comparing the events in five-year periods of the follow-up time. There are no overlapping events, and we used only the baseline data. As expected for a subjective/self-reported measurement, the association is strongest in the beginning; however, SRH still predicted mortality 15–20 years after the question was answered in 1994.

**Table 4:** Self-reported health for the Tromsø 4 cohort without updated values at five-year intervals to examine how its predictive value diminished over time.

		0–5 years			6–10 years			11–15 years			15–21 years		
		HR	LLCI	ULCI	HR	LLCI	ULCI	HR	LLCI	ULCI	HR	LLCI	ULCI
Model 1	**Self-reported health**												
	Poor	4.72	3.02	7.38	2.22	1.72	2.87	1.63	1.17	2.26	2.09	1.61	2.72
	Not so good	2.86	1.90	4.30	2.01	1.65	2.46	1.31	1.05	1.64	1.59	1.35	1.88
	Good	1.55	1.03	2.34	1.53	1.25	1.87	0.98	0.78	1.22	1.25	1.06	1.48
	Very good (baseline)	1.00			1.00			1.00			1.00		
Model 2	**Self-reported health**												
	Poor	3.63	2.30	5.73	1.92	1.48	2.50	1.43	1.02	2.00	1.58	1.21	2.06
	Not so good	2.50	1.66	3.77	1.87	1.52	2.29	1.22	0.97	1.53	1.37	1.16	1.63
	Good	1.50	1.00	2.26	1.51	1.24	1.84	0.97	0.78	1.21	1.18	1.00	1.39
	Very good (baseline)	1.00			1.00			1.00			1.00		

Model 1 is age- and sex-adjusted estimates.

Model 2 is adjusted for all confounders.

HR = hazard ratio, LLCI = Lower level 95% confidence interval, ULCI = Upper level 95% confidence interval.

Table 5 shows the results of hazard ratios of self-reported health by survey and age groups. When comparing the age-sex adjusted model (model 1) and the fully fitted model (model 2), we see that overall HR decreases as expected when controlled for more objective health measures. The confounders had the most substantial effect for the youngest age group, while the attenuation is less pronounced in the oldest age group.

**Table 5:** Results from Cox proportional hazard models of mortality risk of self-reported health stratified by age.

		25–54			55–74			75 or above		
		HR	LLCI	ULCI	HR	LLCI	ULCI	HR	LLCI	ULCI
Model 1	**Self-reported health**									
	Poor	3.11	2.36	4.10	2.44	2.02	2.94	2.38	1.63	3.47
	Not so good	2.01	1.67	2.42	1.82	1.55	2.13	1.90	1.35	2.67
	Good	1.24	1.04	1.48	1.31	1.11	1.53	1.43	1.01	2.01
	Very good (baseline)	1.00			1.00			1.00		
Model 2	**Self-reported health**									
	Poor	2.31	1.73	3.07	2.07	1.71	2.52	2.02	1.37	2.96
	Not so good	1.60	1.32	1.94	1.71	1.46	2.01	1.74	1.23	2.45
	Good	1.09	0.91	1.30	1.28	1.09	1.51	1.44	1.02	2.03
	Very good (baseline)	1.00			1.00			1.00		

Model 1 is age- and sex-adjusted estimates.

Model 2 is adjusted for age, sex, morbidity and risk factors.

HR = hazard ratio, LLCI = Lower level 95% confidence interval, ULCI = Upper level 95% confidence interval.

## Discussion

In this study, we have shown strong relationships between SRH and all-cause mortality over a twenty-five-year follow-up period, with higher SRH levels associated with beneficial hazard ratios. Despite being a subjective and immediate assessment of overall health, SRH was associated with other, more objective health measures. This correlation explains why HRs decrease when controlling for clinical conditions and other risk factors; however, SRH remains an independent predictor of mortality, even when controlling for more objective health measures.

Being an immediate and subjective assessment, one would also expect that SRH at one moment in time would provide information about morbidities, but over time the impact of SRH might be expected to diminish substantially. Attenuation due to time takes place as initial effects are likely to be due to both undiagnosed conditions and coping abilities, which lead to subtle changes in subjective health status.We found that the predictive ability of SRH remained statistically significant over the entire follow-up period, but was attenuated with time. This suggests that although SRH is a subjective and time-dependent instrument, it is still a stable measure over time.

Our study also shows that disease, mental health and subclinical conditions all form part of the ability of SRH to predict mortality. Kaplan *et al*. were the first to describe how the effect of SRH was attenuated when controlling for disease and subclinical conditions. They concluded that SRH levels mainly reflect underlying disease burden, and therefore predict mortality^19^. Later studies have reproduced Kaplan’s results but challenge his conclusion because SRH also reflects coping resources and influences health-related behaviour that affects outcome, and might include a health awareness not captured by the specific health indicators but relevant to a rating of overall health^4,10–17,20,21^.

We see that SRH is not only correlated to known diseases and other objective health measures but also other less easily measured components of health status. The overall pattern of our study suggests that the predictive ability of SRH goes beyond the traditional concept of health as the absence of disease, which implies that SRH is a multidimensional concept incorporating many aspects of health-relevant behaviour for survival. From a health theory perspective, there is a fundamental idea that bodily perceptions can be transformed in response to experiences of illness and health^22,23^. The principal point is that any person will have an overall evaluation of themselves that represents an immediate awareness or response to their circumstances^24^. Some of these experiences are perceived as experiences of health or illness^25,26^.

As suggested by our study, several authors argue strongly that SRH seems to go beyond a model of health as the absence of disease, and thus helps to identify at-risk individuals and illuminate underlying illnesses that may otherwise go undetected during routine examinations of both patients and healthy populations^4,19,20,27^. It is also proposed that SRH reflects comorbidity better than an additive measure of diseases^15,17,27^. SRH is more inclusive than the covariates used in many studies; the idea is that SRH reflects the state of the human organism, which is one reason why SRH is supposed to predict mortality^20^ and why the HRs are supposed to decrease when controlled for objective measures of health status^18^.

Our observation contributes therefore to an ongoing debate on why SRH predicts mortality. Its ability to predict death can hardly imply that SRH has a significant role in a biological causative chain leading to death. The idea is that SRH is a statistical (rather than causative) predictor of mortality because of its ability to reflect the state of the human organism^20^.

Future research should investigate both the logic that governs people’s reasoning about their health and the physiological processes that underlie bodily feelings and sensations. The focus of future research should also be on the pathways that mediate information from the human organism to individual consciousness, thus incorporating that information into self-ratings of health. Lower SRH scores may help identify at-risk individuals and illuminate underlying illnesses that may go otherwise undetected during routine evaluations.

Self-rated health combines culture, psychology, and biology; an interdisciplinary research effort can improve our understanding of SRH as a key measure of health status. We included daily smoking, but future studies should examine the interaction between SRH and health-related behaviours more comprehensively.

### Strengths and limitations

It should be noted that not all participants took part in each survey of the study because Tromsø 5 and 6 included a limited and random selection of participants. Some bias could have been introduced if a change in health status had affected participation during the study period, for example, if those whose health status worsened were less likely to participate in subsequent surveys. The level of non-participation was however not a loss to follow-up due to refusal to take part, but was because people were not invited to subsequent surveys. We therefore regard this as random and unlikely to have resulted in bias. A strength of this study was that follow-up data on the outcome (all-cause mortality) was almost complete for all participants.

It should be noted here that, as in many other studies, self-reported health was assessed using only one general question on the person’s health. This gives a straightforward overall assessment of self-reported health, measured consistently at three time points but does not allow for untangling the relevant dimensions of self-reported health (e.g. physical health compared to mental health) as is possible with self-reported health scores such as the SF-36 and SF-12. We assessed co-morbidity in this study by self-reported diagnosis. Some bias may therefore have been introduced through inaccuracy of reporting. Participant knowledge of comorbidities could have been influenced by factors related to self-reported health such as the severity of the disease, the level of health-seeking behaviour which led to obtaining a diagnosis, and participants’ awareness of their health status influencing the accuracy of reporting comorbidities. While this study was able to adjust for a wide range of health-related factors, it is acknowledged that there are other objective measures of health, e.g. grip strength, where data were not available.

## Conclusion

SRH predicted mortality, but with a time-dependent effect. SRH is affected by disease, mental health and other risk factors, but still predicts mortality independently. We conclude that SRH is still an instrument that has both methodological and clinical value, but its independence from known physical illness and other objective health measures implies that it should not be uncritically utilised as a replacement variable when such measures are lacking.

## Method

### Sample and design

The Tromsø Study is a prospective cohort study consisting of seven repeated population health surveys. They are referred to as Tromsø 1–7 and were carried out in 1974, 1979–80, 1986–87, 1994–95, 2001, 2007–08, and 2015–16. The study includes large, representative samples of the Tromsø population, with the invitation of whole birth cohorts and random samples^28^. The longitudinal design allows us to collect data with the same sample within the Tromsø population. Tromsø 4 was the most extensive survey and included 27158 subjects. Tromsø 4–6 included a second visit with a more extensive examination of the participants. SRH was introduced in the 1986–87 survey; nevertheless, we chose Tromsø 4 as the baseline for two reasons. First, all inhabitants in Tromsø Municipality born before 1970 were invited (the attendance rate was 77%). Tromsø 5 invited all participants who took part in the second visit in Tromsø 4. Tromsø 6 invited all participants from Tromsø 4 for follow-up surveys. Second, Tromsø 3 included SRH but focused on cardiovascular diseases. Although it included 18165 participants, only 14161 of these were followed up in Tromsø 4. Tromsø 3 would therefore have resulted in a smaller cohort and have led to concerns regarding missing variables and attrition. For the survival analysis, we followed all participants in Tromsø 4 (N = 25251) from the day of study entry in 1994 to the date of death or the end of follow-up on 31 December 2017. The subgroups of the cohort are based upon the SRH category they reported. We excluded subjects who had missing values for SRH (n = 40) or any of the other variables at baseline (n = 884). Thus, we included 24309 participants (52% women) aged 25–97 years at baseline for the analysis.

We updated SRH and risk factor values at the time of examination for all subjects who attended again in Tromsø 5 (n = 6808) and Tromsø 6 (n = 9313).

The Regional Committee for Medical and Health Research Ethics (REC) and the Norwegian Centre for Research Data (NSD) have approved the Tromsø Study. The study was performed in accordance with relevant guidelines and regulations (i.e. the Helsinki Declaration, Vancouver Convention, and Norwegian legislation). Informed consent was obtained from all participants.

### Measurements

The participants completed well-validated surveys that included questions on a broad range of diseases, symptoms, health behaviour, social conditions, education, and level of physical activity. The conceptual model was that SRH predicts all-cause mortality and that this effect can vary with sex, age, comorbidity, and other known risk factors. All-cause mortality is the outcome of interest, while SRH was the independent variable of interest. We treated the other variables as confounders for the sake of the presentation.

We retrieved the time of death from the Norwegian National Cause of Death Registry. The degree of coverage of the registry is near-complete^29^. SRH was reported by answering the survey question ‘What is your current state of health?’ with answers ranging from very poor (1) to very good (5) in Tromsø 3 and 6, and ranging from poor (1) to very good (4) in Tromsø 4 and 5. Since less than 0.37% of the participants rated their health as very poor, we merged poor and very poor for the sake of the analysis.

Specially trained personnel took non-fasting blood samples and measured blood pressure (SBP/DBP), cholesterol, body weight and height. Comorbid diseases were self-reported specific medical conditions. We selected 13 symptomatic medical conditions known to be associated with SRH and mortality: myocardial infarction, angina, cerebrovascular stroke, diabetes, bronchitis, asthma, duodenal and gastric ulcer, arthrosis, osteoporosis, psoriasis, migraine, and thyroid disease. The severity of the disease affects the level of SRH. Therefore, we used the Health Impact Index (HII) to measure the comorbid conditions. HII classifies individuals with a comorbid disease according to the impact that each condition has on SRH by assigning a weight to each condition. The HII represents the subject’s total score for all conditions; thus, HII considers both the severity and joint effects of conditions^30^. We have added supplementary information to show the association between these diagnoses and SRH.

Mental health symptoms were based on well-validated self-report symptom inventories comprising questions representative of the symptom configurations of anxiety and depression commonly observed among outpatients. Each answer is scored from 1 to 4. The measurement is the average score. The mental health index (CONOR-MHI) was used at TS4. In the following surveys (TS5–6), the Hopkins Symptom Checklist (HSCL) was used (13). The CONOR-MHI has been compared with the HSCL with reasonably good agreement. It was highly correlated with HSCL-10 (r = 0.8). A cut-off level of 2.15 for significant symptoms is equivalent to the 1.85 level in HSCL-10 (14).

Hypertension is defined as having systolic blood pressure above 140 mmHg, diastolic blood pressure above 90 mmHg, or being on active treatment. Hyperlipidaemia is defined as having either total cholesterol levels above 8 mmol/l, Low-density lipoprotein cholesterol (LDL) above 5 mmol/l or being on active treatment.

### Statistical analysis

We used STATA v15 for all statistical analysis. Follow-up time was person-age. Subjects entered and exited at their age measured in days. Time extended from the date of study entry to the date of death, or end of follow-up on 31 December 2017. We used Cox proportional hazard regression to estimate HRs of death using scores for exact age in days at the first survey in which participants took part in addition to SRH, BMI, comorbid disease, and physical examination scores as time-dependent covariates. All scores were updated in 2001 and 2007/8 for those who participated. The primary model in Table 3 includes repeated measures of SRH in an attempt to capture changes in self-reported health over time.

SHR is not necessarily a stable measure and may change over time. It is reasonable to assume that over time its impact would diminish to a considerable degree. We wanted to examine how SRH at one moment in time can be very informative, but as time goes on, it becomes less relevant to subsequent health status. We therefore repeated the analysis above without updating SRH from the first survey. We also checked SRH without updated values at five-year intervals to examine how its predictive value diminished over time. The results are presented in Table 4.

The proportional hazard assumption was verified by visual inspection of log-log survival curves and by tests of Schoenfeld residuals. The visual inspection and the Schoenfeld test indicated that the proportional hazard assumptions were not violated.

Public health has changed in Norway during the time of observation. We wanted to examine whether the associations remained stable during the period or whether the changes could be a cohort effect. We therefore stratified the cohort by age at baseline (25–54 years, 55–74 years, and 75 or above). Model 1 is age- and sex-adjusted HR, while model 2 includes all covariates. Very good was the baseline category.

## Supplementary information

Health impact index.

## Data Availability

Data is available from the Tromsø Study for researchers who meet the criteria for access to confidential data (https://en.uit.no/prosjekter/prosjekt?p_document_id=80172). Readers may also contact Professor Sameline Grimsgaard sameline.grimsgaard@uit.no, to request the data or receive a confirmation that data will be available upon request to all interested researchers. Furthermore, all variables are described in the NESSTAR database. http://tromsoundersokelsen.uit.no/webview/.
