# Supplementary material for: Self-reported health as a predictor of mortality: A cohort study of its relation to other health measurements and observation time
Source: Sci Rep. 2020 Mar 17;10:4886. doi: 10.1038/s41598-020-61603-0 (PMC7078209; doi:10.1038/s41598-020-61603-0)
Supplement: Supplementary file 1 — Health impact index. [file 41598_2020_61603_MOESM1_ESM.pdf]

Self-reported health as a predictor of mortality: A cohort study of its relation to other health measurements and observation time

Professor Geir Lorem[1], Dr Sarah Cook[2], Professor David Leon[2], Professor Nina Emaus[1], Professor Henrik Schirmer[1,3,4]

[1]UiT The Arctic University of Norway

[2] London School of Hygiene & Tropical Medicine

[3]University of Oslo

[4]Akershus University Hospital

Corresponding Author:

Geir Fagerjord Lorem

Department of Health and Care Sciences

Faculty of Health Sciences, UiT The Arctic University of Norway

Geir.lorem@uit.no

Diagram and table showing the associations between the 13 selected medical conditions and self-reported health

We utilised the Health Impact Index (HII) to measure the comorbid conditions. It is an instrument that accounts for both the severity and joint effects of the diseases compared to self-reported health.[1] The conditions were self-reported by answering survey questions such as "Do you have or have you had....?" We classified each participant for each known diagnosis. All conditions are listed in Figure 1. Only participants older than 70 years answered questions about Parkinson's disease and arthritis.

We used SRH from Tromsø 4 as the outcome of interest when estimating the association between the comorbid diseases and SRH. We used ordinal logistic regression to assess the relationship between the diseases and SRH in the baseline population. The coefficients are the probability of scoring at higher levels of SRH for those with the disease compared with those without the disease. A coefficient lower than zero is thus associated with a probable negative impact on SRH.

Figure 1 and Table 1 show the association between the 13 selected medical conditions and self-reported health.

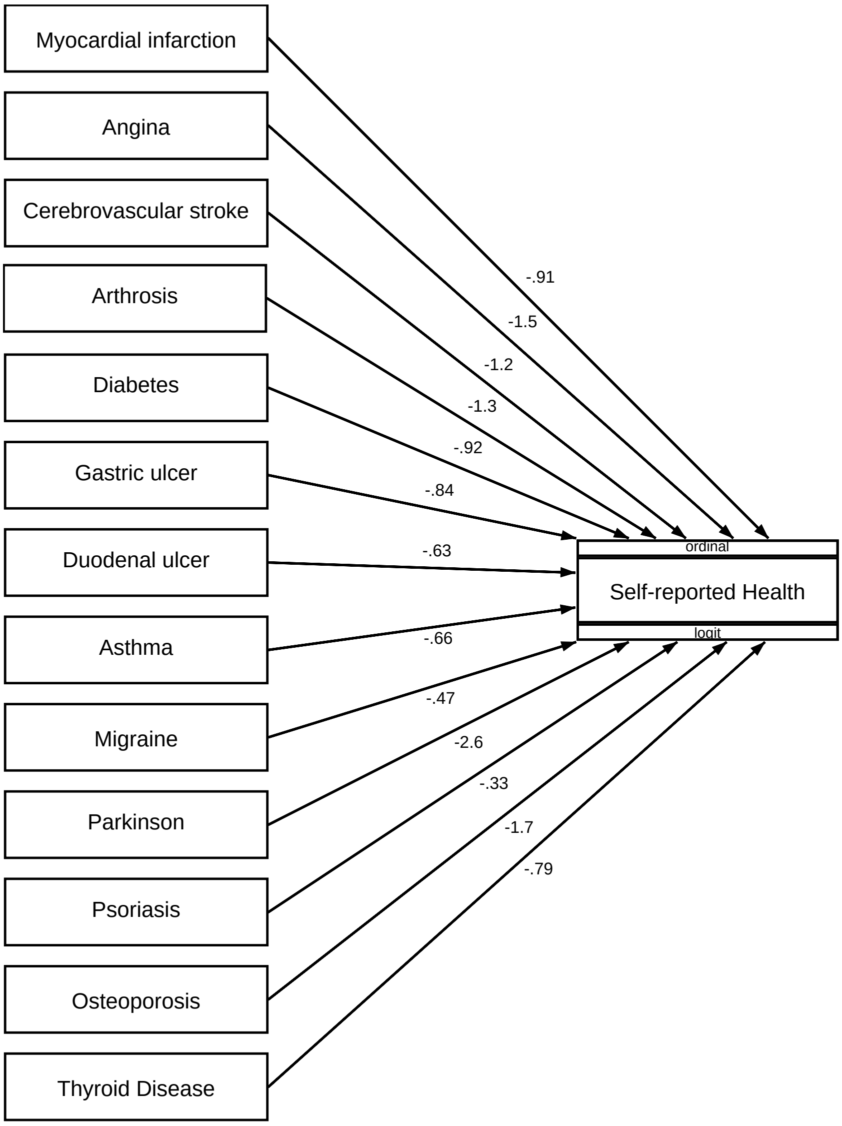

Figure 1. The DAG diagram for the association between the self-reported diagnosis and SRH

Table 1. The results of the ordinal logistic regression model

	Coef.	Std. Err.	z	P>z	[95% Conf.	Interval]
Myocardial infarction	-.9117221	.106264	-8.58	<0.001	-1.119996	-.7034485
Angina	-1.503473	.092414	-16.27	<0.001	-1.684601	-1.322345
Stroke	-1.196207	.1410485	-8.48	<0.001	-1.472657	-.9197571
Diabetes	-.9232748	.1204793	-7.66	<0.001	-1.15941	-.6871398
Gastric ulcer	-.8373743	.0796475	-10.51	<0.001	-.9934805	-.6812681
Duodenal ulcer	-.6258091	.0836911	-7.48	<0.001	-.7898406	-.4617776
Asthma	-.6582203	.0586318	-11.23	<0.001	-.7731365	-.5433041
Migraine	-.4694137	.0418954	-11.20	<0.001	-.5515271	-.3873002
Parkinson	-2.590215	.6207989	-4.17	<0.001	-3.806959	-1.373472
Psoriasis	-.3348447	.0568435	-5.89	<0.001	-.4462559	-.2234334
Osteoporosis	-1.665954	.1425084	-11.69	<0.001	-1.945266	-1.386643
Thyroid disease	-.7938499	.0841311	-9.44	<0.001	-.9587438	-.6289561
Arthrosis	-1.286765	.0993391	-12.95	<0.001	-1.481466	-1.092064
**Self-reported health**						
cut1	-4.845064	.0649312			-4.972327	-4.717801
cut2	-1.485834	.0209043			-1.526806	-1.444863
cut3	1.399039	.0207642			1.358342	1.439736

Number of obs = 19,224, Response: Self-reported health, Family: Ordinal, Log likelihood = -18581.326

Reference

1 Lorem, G. F., Schirmer, H. & Emaus, N. Health Impact Index. Development and validation of a method for classifying comorbid disease measured against self-reported health. *PloS One* **11**, doi:http://dx.doi.org10.1371/journal.pone.0148830 (2016).
